# Perceptions of an automated benchmarking dashboard for antimicrobial stewardship programs among antimicrobial stewards within the veterans’ health administration: a multicenter qualitative study

**DOI:** 10.1017/ash.2023.203

**Published:** 2023-07-10

**Authors:** DeShauna Jones, Alexandre R. Marra, Daniel Livorsi, Eli Perencevich, Michihiko Goto

**Affiliations:** 1 Center for Access & Delivery Research and Evaluation (CADRE), Iowa City VA Health Care System, Iowa City, IA, USA; 2 University of Iowa, Institute for Clinical and Translational Science, Iowa City, IA, USA; 3 University of Iowa, Carver College of Medicine, Iowa City, IA, USA; 4 Hospital Israelita Albert Einstein, São Paulo, SP, Brazil

## Abstract

**Objective:**

To evaluate the impact of a multicenter, try automated dashboard on ASP activities and its acceptance among ASP leaders.

**Design:**

Frontline stewards were asked to participate in semi-structured interviews before and after implementation of a web-based ASP information dashboard providing risk-adjusted benchmarking, longitudinal trends, and analysis of antimicrobial usage patterns at each facility.

**Setting:**

The study was performed at Iowa City VA Health Care System.

**Participants:**

ASP team members from nine medical centers in the VA Midwest Health Care Network (VISN 23).

**Methods:**

Semi-structured interviews were conducted pre- and post-implementation, with interview guides informed by clinical experiences and the Consolidated Framework for Implementation Research (CFIR). Participants evaluated the dashboard’s ease of use, applicability to ongoing ASP activities, perceived validity and reliability, and relative advantage over other ASP monitoring systems.

**Results:**

Compared to established stewardship data collection and reporting methods, participants found the dashboard more intuitive and accessible, allowing them to reduce dependence on other systems and staff to obtain and share data. Standardized and risk-adjusted rankings were largely accepted as a valuable benchmarking method; however, participants felt their facility’s characteristics significantly influenced the rankings’ validity. Participants recognized staffing, training, and uncertainty with using the dashboard as an intervention tool as barriers to consistent and comprehensive dashboard implementation.

**Conclusions:**

Participants generally accepted the dashboard’s risk-adjusted metrics and appreciated its usability. While creating automated tools to rigorously benchmark antimicrobial use across hospitals can be helpful, the displayed metrics require further validation, and the longitudinal utility of the dashboard warrants additional study.

## Background

Antimicrobial resistance (AMR) is an urgent public health threat globally and in the US.^1,2^ In 2013, the Centers for Diseases Control and Prevention estimated that infections with AMR were associated with at least 23,000 excessive deaths annually in the US.^3^ Antimicrobial stewardship programs (ASPs) play vital roles in reducing unnecessary antimicrobial use and combating AMR. One of the key recommendations for ASPs is to measure antimicrobial consumption as a metric.^4^

To be effective, ASPs need practical methods for monitoring antimicrobial use.^4–6^ In 2014, the Veterans Health Administration (VHA) mandated all its facilities develop and maintain an ASP.^7,8^ However, according to a 2015 mandatory survey of 140 VA facilities, 64% reported that limited IT support and/or data tools presented “substantive challenges to achieving optimal antimicrobial use.”^9^

To address this critical resource gap, we developed a multicenter, automated electronic dashboard for ASPs that displays risk-adjusted benchmarking metrics for different categories of antimicrobial consumption. This dashboard was deployed in the acute care, intensive care, and long-term care units of 10 VHA hospitals that participated as pilot users. This study aimed to evaluate the impact of these metrics on ASP activities and the acceptance of these metrics among ASP members.

## Methods

### Ethics

The Institutional Review Board of the University of Iowa and Iowa City Veterans’ Health Care System approved this study. A waiver for written informed consent was granted.

### Setting

VA Midwest Health Care Network (VISN 23) serves over 440,000 enrolled Veterans through an integrated system of 10 acute-care medical centers and eight long-term care facilities, ie, community living centers (CLCs). Additionally, VISN 23 has 69 community-based outpatient or outreach clinics and four domiciliary residential rehabilitation treatment programs, but these were not covered by the implemented dashboard. Hospital capacity at each site ranges from 15 to 229 beds, while CLC size is up to 225 beds. VISN 23 also includes special programs such as a spinal cord injury (SCI) program, cardiac surgery, polytrauma, and transplant.

The dashboard development team is located at the Iowa City VA Health Care System and collaborated with antimicrobial stewards at all 10 medical centers. All facilities designated physician and pharmacist champions for ASPs according to the VHA internal guideline, but the availability of local expertise, especially infectious diseases specialists and informatics support, varied. Specifically, four medical centers did not have any local infectious diseases specialist, and only two facilities had pharmacists who specialize in infectious diseases. No facility had informatics support specifically allocated for ASPs.

### Dashboard tool

We built a system to extract patient-level data for antimicrobial consumption and demographics for acute inpatient and long-term care units at all VHA hospitals each month, utilizing the VHA’s Corporate Data Warehouse. We also collected data for underlying diagnoses prior to hospital admissions and procedures 90 days prior to or during admission. Underlying diagnoses were obtained from inpatient and outpatient diagnoses (International Classification of Diseases 10th edition [ICD-10]) and were classified into 86 categories based on Hierarchical Condition Categories.^10^ Procedures were obtained from inpatient and outpatient procedure records recorded as ICD-10 procedure codes or Current Procedural Terminology codes. Those codes were classified into 224 categories based on Clinical Classifications Software developed by the Agency for Healthcare Research and Quality.^11^ We performed risk adjustments based on negative binomial regression models with patient- and unit-level factors by calculating observed-to-expected ratios of antimicrobial use for each hospital and for specific units within each hospital.

Risk adjustment models included month, unit types (eg, intensive care unit [ICU] vs non-ICU for acute care), specialty, age, gender, comorbidities (50 and 30 factors for acute care and long-term care, respectively), and preceding procedures (45 and 24 procedures for acute care and long-term care, respectively). We created additional models for each antimicrobial category based on National Healthcare Safety Network (NHSN) definitions. For each hospital, risk-adjusted benchmarking metrics and a monthly ranking within the VHA system were visualized and presented interactively to end users through the dashboard (Supplementary Material).

Each hospital had its dedicated dashboard, and the access was restricted only to participating ASP stewards and antimicrobial prescribers. The technical detail of the dashboard has been described elsewhere.^12^

### Design

The Principal Investigator (M.G.) identified frontline ASP stewards and antimicrobial prescribers from all 10 VISN 23 sites to participate in semi-structured interviews pre and post Center for Antimicrobial Stewardship and Prevention of Antimicrobial Resistance (CASPAR) dashboard implementation with the goal of interviewing at least one stewardship team member from each site. A qualitative trained medical sociologist (D.J.) created both the pre- and post-implementation interview guides informed by the clinical experiences of M.G. and D.L. and structured by the Consolidated Framework for Implementation Research (CFIR).^13^ Two of five CFIR domains were of central focus to this research: the inner setting and intervention characteristics.

After completion of preimplementation interviews, we held a presentation and a live demonstration of the dashboard, then provided participants access to the dashboard. Two months following the initial presentation and the deployment of the dashboard to VISN 23, we recruited ASP champions and antimicrobial prescribers for post-implementation interviews from the live demonstration electronic mailing list. Participants were asked to evaluate several aspects of the dashboard including its ease of use, applicability to ongoing ASP activities, perceived validity and reliability, and advantages compared to other ASP monitoring systems. Additionally, all participants were asked to suggest potential participants in VISN 23 with stewardship experience or knowledge of stewardship metrics who could serve as participants.

### Data and analysis

All interviews were conducted by D.J. via audio or videoconference call and were recorded. Interviews were transcribed and deidentified by an internal team of transcriptionists and D.J. reviewed the transcripts for accuracy and completeness.

Cleaned transcripts were uploaded into MaxQDA 2020.4 (VERBI Software, Berlin, Germany). D.J. employed a deductive approach to analysis, using the interview guide and the two CFIR domains of central focus to this study to identify barriers and facilitators associated with the CASPAR dashboard (intervention characteristics) and the participants’ facility (inner setting). An inductive approach to the data followed, identifying central themes across codes.

## Results

We completed four preimplementation interviews and 11 post-implementation interviews from nine VISN 23 VA healthcare systems representing diverse perspectives of ASPs: ten pharmacists, four infectious disease physicians, one pharmacy program manager, and one pharmacy executive. Only one participant completed both a preimplementation and post-implementation interview, and two interviews were conducted wherein two ASP members from one facility were interviewed simultaneously. Three facilities were represented by more than one participant.

Four themes were identified throughout pre- and post-implementation interviews related to the adoption of the CASPAR dashboard: (1) the dashboard improved efficiency of established data collection practices, (2) stewards generally accepted the standardized risk-adjusted metrics as potential benchmarks, but facility size and structure shaped perceived utility of the dashboard, (3) additional training and staff was suggested to facilitate dashboard adoption, and (4) some stewards expressed uncertainty surrounding the dashboard’s ability to inform stewardship intervention efforts.

### Theme 1. The CASPAR dashboard addressed less efficient established data collection and reporting systems

During pre- and post- interviews, participants were asked to describe how they obtained antimicrobial prescription data, with whom they shared the data, and how they used this data to inform stewardship activities. Most reported using data from the NHSN healthcare-associated infection tracking system and expressed difficulty finding essential data within this system, often relying on other staff to locate information. In contrast, the interface of the CASPAR dashboard was described as “easier to get around in,” “responsive,” and “user-friendly” (Table 1). The dashboard’s “useful data” and graphics were described as easier to incorporate into reports and presentations which facilitated communication of ASP metrics across facilities.

**Table 1.** CASPAR dashboard improves data collection

Description *[CFIR Domain; subdomain]*	Verbatim Quotes
Ease of dashboard use *[Intervention Characteristics; design quality and packaging, relative advantage]*	“It’s very dependable is what I would say. Comparatively to other dashboards…and-and I find this much easier to get around in than maybe the NSHN pieces, etc.” (Pharmacist, site 8).
	“We generally had NHSN data presented to use by members of our stewardship team. It’s been relatively hard for me to access that on my own…It’s been nice to have this to be able to look at on my own time” (Pharmacist, site 4).
	“I usually report quarterly data to the P & T Committee that has both NHSN and then a big portion of it now is the CASPAR dashboard which is a lot easier to put in and to use the slides in my quarterly reports that I use for that. So that’s been very useful to me. Our NHSN data you know we’ve had to rely on the VISN 23, with (name) and her group, you know getting that data in, so that can be delayed. But here, I can always go in and get access to the CASPAR data immediately at any point in time, I think I could look at things. I really like that accessibility without having to wait on someone else” (Pharmacist, site 8).
	“…it’s readily available with the bookmark and that I don’t have to um, contact one of our data analysts to say, ‘Hey can you, can you pull this data out to present it at my next meeting?’ So, it’s ready to review when I have time to do so. That’s, that’s very nice. And it’s fairly user friendly and intuitive with the caveat that I, I should um, I should get better at using it and getting more familiar with it” (Physician, site 3).
Dashboard improves collection of CLC and outpatient data *[Intervention Characteristics; design quality & packaging, relative advantage]*	“Another one of the really nice things about the dashboard–it has a CLC dashboard and being able to get data for the CLC unit has been very challenging, so that was nice” (Pharmacist, site 5).
	“Because the providers are free texting in what their indication is, you might be looking for UTIs, but some people might put UTI. Some people might put cystitis. Some might put urinary tract infection. So, it’s not easy to pull that up in a spreadsheet. You’re still doing a lot of data mining. Then it makes it more difficult, I think, to get meaningful information from that” (Pharmacist, site 2).
	“As I’ve mentioned didn’t really have a way to track the CLCs, so that’s a huge improvement right there., so originally, I was using more like the NHSN reports and their SAAR data and those reports are cumbersome and would take time to like pull the data out that you needed. Then I was using the dashboards that I believe (name) in charge of how, –which was nice cause then it would compare our facility to other VA facilities, um, but I believe they’re having some issues with those dashboards, because I don’t think they’ve been updated since January. This dashboard is just so much more user friendly, um, it can get the information quickly, it’s all right there. It has nice pretty pictures, it’s um much easier to use and I also like how it compares you to all of the VAs with their risk stratification which is nice” (Pharmacist, site 5).

In addition to its user-friendly interface, the CASPAR dashboard reportedly reduced the amount of time spent working with multiple systems and staff. Concerning established data collection systems, participants described querying several systems, making stewardship “a little bit messier.” In the words of one physician, the ASP team felt they were, “…not able to focus on actually doing interventions with that data, we’re just trying to *get* the data” (site 4). In contrast, a pharmacist remarked that the CASPAR dashboard allowed access to data “immediately, at any point in time” without waiting for other personnel to update or retrieve data (site 8).

Participants also felt CASPAR metrics were more complete and accurate than NHSN metrics. The ability to track antibiotic usage in CLCs using the CASPAR dashboard was viewed as a “huge improvement” and addressed participants’ prior difficulties obtaining CLC data (pharmacist, site 5). Furthermore, the ability of dashboard metrics to adjust for “diagnostics, demographics, comorbidities, all different categories” unavailable in NHSN enhanced perceptions of the metrics’ validity (pharmacist, site 4).

### Theme 2. Stewards largely accepted standardized and risk-adjusted metrics as potential benchmarks, but facility size and structure shaped perceived utility

Due to a lack of mutually accepted benchmarks within antibiotic stewardship practice, participants felt risk-adjusted metrics were an acceptable way to compare facilities and set benchmarks to assist with goal setting. However, during preimplementation interviews, participants reported NHSN hospital rankings as ineffective for goal setting due to their inability to adjust for significant facility characteristics. After reviewing CASPAR rankings, several participants regarded them as a superior metric. As one physician indicated, the NHSN dashboard “compares hospitals after adjusting for just a small number of factors such as the size of the hospital, whether the hospital has an ICU, and whether it’s a teaching hospital. So far, far fewer factors than [the CASPAR] dashboard is accounting for” (site 7). However, some participants expressed hesitancy to fully adopt the rankings as a benchmarking method due to concerns about the ability of risk adjustments to account for facility differences (Table 2).

**Table 2.** Perspectives of standardized and risk-adjusted rankings as benchmarks

Description *[CFIR Domain; subdomain]*	Verbatim Quotes
Acceptance of rankings as benchmarks *[Intervention Characteristics; design quality and packaging, relative advantage]*	“I appreciate that the data represented in the dashboard allows us to compare to other facilities our same size or compare to other facilities that offer similar services to us so that we are better able to determine benchmarks if you will with similar kind of facilities rather than just VISN data because the other facilities in our VISN are quite different from us with respect to antimicrobial use” (Pharmacy executive, site 9).
	“I think it gives us a benchmark and as we work on process improvement and we can see that change in comparison to our current ranking It might not be 100% accurate, but we can still monitor trends I guess” (Pharmacy manager, site 6).
Facility size and structure shape acceptance *[Inner Setting; structural characteristics, Intervention Characteristics; design quality and packaging, relative advantage]*	“I really like it, I think it gives at least to a smaller facility better looking data because we could have one patient that can skew our results, quite a bit when I look at the NHSN data, especially on the SAAR numbers and things, during COVID for sure they just went sky high and there were a few patients that stayed in there for a long time and things didn’t go as planned, but now after that is gone, that number has come down” (Pharmacist, site 8).
	“VISN 23 there are some hospitals that are always gonna remain a smaller, community hospital because, the population in that area is smaller…but the problem with all of these is that, even when you control for size, the amount of improvement you can bring about may never be enough to displace some of the smaller hospitals where, if you reduce the antibiotic script by five, you can leap frog from position number three to position number one” (Physician, site 3).
	“We have a quite large CLC, and there’s no other facility in our VISN that quite compare size-wise. And so, looking at like among the other comparative hospitals where we stand, those areas, it’s more helpful to us because across our VISN we share some data and have some dashboards and things that have been created, specifically for, –by our group but we tend to be a little bit of a different facility that doesn’t, –it’s like comparing apples and oranges sometimes” (Pharmacy manager, site 6).

Participants’ perspectives of their facility’s attributes, including size, structure, and patient population, influenced the perceived value of hospital rankings. Some participants felt CASPAR hospital rankings penalized their facility for its size and patient population. For example, a pharmacy manager from a small facility remarked, “We have to keep in mind that we have a very small sample size that can throw off these [rankings]” (site 6). Similarly, participants from facilities taking in more critically ill patients felt that their rankings would “look worse than they really are” (physician, site 7). Still, other participants felt the hospital rankings did not address a larger theoretical pitfall within the stewardship world:

*“I think one of the biggest things that we all struggle with is how do we understand the appropriate level of use? Which I know is a kind of a philosophical part that’s not really a fix the dashboard can give, but I think we all look at this and we wonder, ‘Okay, here we are, we’re, you know, at 25% of facilities here. Is that lower than we want to be? Higher than we want to be?’” (physician, site 3).*

### Theme 3. Additional training and staff are needed to facilitate CASPAR dashboard adoption

While participants generally reported the CASPAR dashboard increased the efficiency of collecting and reporting data and presented more complete and accurate data, participants felt staff shortages presented challenges to learning how to navigate a new system:

*“…it likely starts with staffing where one or two dedicated personnel have to be assigned to this. It’s more like one person a week, a new person picks up the next week. So, there is the lack of continuity and if we can address that I think that would be the first step towards obtaining the data and sharing it” (physician, site 3).*

One participant viewed the CASPAR dashboard as a potential tool to advocate for more staff and additional stewardship resources (Table 3).

**Table 3.** Additional resources needed to fully adopt dashboard

Description *[CFIR Domain; subdomain]*	Verbatim Quotes
Staff shortages challenge adoption *[Inner setting; readiness for implementation-available resources*]	“If we’re looking at process improvement, and it could be incorporated into some of the metrics that our quality team is monitoring, that would be awesome because I think it would be more feasible and sustainable if it could be incorporated into the routine quality workflow versus having a pharmacist that doesn’t have FTE set aside to be doing these sorts of things, trying to fit it in with the other workflow” (Pharmacy manager, site 6).
	“I think we’re really low staffed. Certainly, you know we could also use more people, but I think we are doing a wonderful job with the people that we have and everybody’s very engaged” (Physician, site 4).
	“I know that we maybe haven’t always been as forward thinking in this VISN with stewardship, haven’t always staffed every facility the way we needed to, so the more data we have, the better we could advocate and defend the need that we have for people, for systems, etc.” (pharmacist, site 3).
	“We don’t really have a dedicated person that their only job is ASP. It’s kind of part of a lot of people’s jobs” (pharmacist, site 2).
Training needs *[Inner setting; Readiness for implementation-access to knowledge and information]*	“I just need to know how to use it a little bit better and like I said like a little legend or a key, or a how to guide” (pharmacist, site 3).

### Theme 4: Some stewards expressed uncertainty surrounding how CASPAR informs specific stewardship interventions

When asked what additional functionalities would be useful to incorporate into the dashboard, some respondents reported that rather than adding more metrics, they desired more direction on how to use the metrics to actively *improve* antimicrobial prescribing at their facility rather than using the dashboard as an evaluative tool for antimicrobial prescribing that already occurred (Table 4). One physician stated, “It just sort of provides a general overall assessment, but it doesn’t tell you exactly how you can improve aside from just decreasing antibiotic use—which is less actionable than some other hospital metrics” (site 7). In a similar vein, a pharmacy manager described the CASPAR dashboard as, “…really interesting—a lot of comparative data that’s very helpful, I just have a hard time trying to use the data and convert that into a measurable process improvement initiative” (site 6).

**Table 4.** Using the dashboard as an intervention tool

Description *[CFIR Domain; subdomain]*	Verbatim Quotes
Desire to use CASPAR data to develop stewardship interventions *[Inner setting; Implementation climate-compatibility]*	“They’re not really telling us where the options for improvement are. They’re just kinda saying, oh, your antibiotic use in general is too high or your use of this specific agent is too high but that’s not necessarily how clinicians think. They don’t think “oh, I’m using too much Vancomycin, and think about okay, but here’s a patient with pneumonia in front of me, what’s the best way to treat this patient?” um, acknowledging that I wanna treat whatever bugs are likely to be causing this infection but also I don’t want to give antibiotics that are unnecessarily broad. So something that’s more in line with how a clinicians approach patient care, which would probably be more syndrome based would be more actionable and would be more useful to our program, but that’s not to say that this type of approach is not useful to programs that have a large hospital and you know they aren’t able to kinda keep tabs on things as closely as we are, being a small facility. But for us specifically a dashboard that was more syndrome based, um, would be more helpful” (Physician, site 7).
	“I think just examples of how it’s going to be utilized and workflow at other facilities and some suggestions on what to do with all the information” (Pharmacy manager, site 6).

## Discussion

ASP leaders desire metrics to assess the impact of stewardship activities in a variety of healthcare settings;^14,15^ however, the effort required to extract metrics from electronic health records and translate analysis into tangible interventions are technically challenging and often times impossible with available resources. By combining multidisciplinary expertise (infectious diseases, pharmacy, informatics, and qualitative evaluation), we leveraged electronic medical record data to operationalize a centralized dashboard displaying risk-adjusted, hospital-level antimicrobial use. Through semi-structured interviews conducted before and after dashboard implementation, we identified several attributes that ASP leaders appreciated: a user-friendly interface, antimicrobial surveillance data for long-term care facilities (i.e., CLCs), and more rigorous hospital rankings on antimicrobial use. We demonstrated that there is a relative advantage in our risk-adjusted ASP metrics compared to currently available tools for benchmarking, eg, NHSN. Evaluating performance data facilitates hospital surveillance of defined and consistent metrics to ensure continuous improvement across different settings included in the dashboard.^16^

Like other studies focusing on user acceptance of digital interventions for antimicrobial prescribers and stewards,^15,17,18^ we found that although the dashboard released stewards from intensive data gathering, potentially leaving more time for developing appropriate interventions, some stewards expressed distrust of the dashboard metrics. Stewardship team members in facilities larger or smaller than average or caring for more critically ill patients expressed apprehension in accepting dashboard rankings. Additionally, while the dashboard reduced the burden of low staffing during the data collection phase, the difficulty of securing time to learn the new dashboard more thoroughly increased and did not completely resolve the well-documented short staffing of ASPs.^19–21^ Finally, and perhaps most significantly, some stewards voiced concern of a larger theoretical gap surrounding the interpretation of interfacility rankings and how the dashboard metrics could inform future interventions.

Our work attends to the need to “develop and validate metrics to guide more comprehensive evaluations of antimicrobial-prescribing at the facility-level.”^22^ Additional work is needed to evaluate the validity of comparing hospitals using the dashboard’s risk-adjusted version of a standard antimicrobial consumption metric. Furthermore, it remains unclear how the dashboard is being used longitudinally and what the consequences of this use are. Considering these needs, we see the work discussed in this report as an initial step to designing and implementing the dashboard on a broader scale.

Our study has both strengths and limitations. This qualitative study represents one of few studies to assess the acceptance of an automated dashboard tool for evaluating antimicrobial stewardship performance. Since all sites were VA hospitals, our findings may be less relevant to non-VA and non-US facilities. We chose sites with diverse care settings; however, the barriers described were common across sites. Furthermore, while the sample size (n = 15) is relatively small, the purposive sampling strategy and sample size were appropriate choices given the small sampling pool. Additionally, only one participant completed both a pre- and post-implementation interview, and we did not assess perspectives of prescribers not involved in leading ASP activities. Interviewees self-reported their processes, and we did not validate the accuracy of their statements. Furthermore, although interviews were confidential, participants may have been inclined to give socially desirable responses.

Given the limited resources for antimicrobial stewardship personnel, electronic tools in antimicrobial stewardship are an attractive method to facilitate compliance and improve efficiency.^23,24^ Emerging information technology is now opening the door to objective assessment of programs by peer-to-peer comparison (benchmarking). This, in turn, allows limited stewardship resources to be allocated for other stewardship activities. Our experience with the development and deployment of a dashboard tool demonstrates a large potential for informatic tools to facilitate antimicrobial surveillance and benchmarking, but the concurrent training and technical supports, as well as transparency for the data collection and risk adjustment, are important to achieve acceptance among ASP leaders.

## Supplementary material

The supplementary material for this article can be found at https://doi.org/10.1017/ash.2023.203
